# RNase E-dependent degradation of *tnaA* mRNA encoding tryptophanase is prerequisite for the induction of acid resistance in *Escherichia coli*

**DOI:** 10.1038/s41598-020-63981-x

**Published:** 2020-04-28

**Authors:** Takeshi Kanda, Genta Abiko, Yu Kanesaki, Hirofumi Yoshikawa, Noritaka Iwai, Masaaki Wachi

**Affiliations:** 1 Department of Life Science and Technology, Tokyo Institute of Technology, 4259 Nagatsuta, Midori-ku, Yokohama, 226-8501 Japan; 2 NODAI Genome Research Center, Tokyo University of Agriculture, 1-1-1 Sakuragaoka, Setagaya-ku, Tokyo, 156-8502 Japan; 3 Present Address: Research Institute of Green Science and Technology, Shizuoka University, 836 Ohya, Suruga-ku, Shizuoka, 422-8529 Japan; 4 Department of Bioscience, Tokyo University of Agriculture, 1-1-1 Sakuragaoka, Setagaya-ku, Tokyo, 156-8502 Japan

**Keywords:** Bacteriology, Pathogens

## Abstract

Acid-resistance systems are essential for pathogenic *Escherichia coli* to survive in the strongly acidic environment of the human stomach (pH < 2.5). Among these, the glutamic acid decarboxylase (GAD) system is the most effective. However, the precise mechanism of GAD induction is unknown. We previously reported that a *tolC* mutant lacking the TolC outer membrane channel was defective in GAD induction. Here, we show that indole, a substrate of TolC-dependent efflux pumps and produced by the tryptophanase encoded by the *tnaA* gene, negatively regulates GAD expression. GAD expression was restored by deleting *tnaA* in the *tolC* mutant; in wild-type *E. coli*, it was suppressed by adding indole to the growth medium. RNA-sequencing revealed that *tnaA* mRNA levels drastically decreased upon exposure to moderately acidic conditions (pH 5.5). This decrease was suppressed by RNase E deficiency. Collectively, our results demonstrate that the RNase E-dependent degradation of *tnaA* mRNA is accelerated upon acid exposure, which decreases intracellular indole concentrations and triggers GAD induction.

## Introduction

Pathogenic *Escherichia coli* strains cause severe illness, such as enteric and diarrheal diseases^1,2^. As they have low infectious doses and are transmitted through ubiquitous media, including food and water, such outbreaks are serious public health concerns in both developing and developed countries^1^. In developing countries in sub-Saharan Africa and South Asia, pathogenic *E. coli* is a major contributor to diarrheal disease, particularly in children under 5 years of age^3^. In Japan, the largest outbreak of enterohemorrhagic *E. coli* O157:H7, which occurred in primary schools in 1996, caused >7,500 reported infections and three deaths^4,5^. The most serious outbreak in Germany in 2011 was caused by Shiga toxin-producing *E. coli* O104:H4, which caused 3,816 infection cases and 54 deaths^6^.

Although pathogenic *E. coli* are classified into several categories based on unique features of their interactions with host cells^7^, infection is universally established by colonization in the intestine, mainly through oral transmission^1,8^. Pathogenic *E. coli* are inevitably exposed to the extremely acidic stomach environment, which acts as a primary bactericidal barrier^9,10^. To overcome this acidic stress, *E. coli* (both pathogenic and non-pathogenic strains) possess sophisticated acid-resistance (AR) systems. These systems and their complex regulation have been extensively described in recent reviews^11–13^. Five primary systems, designated AR1–AR5, have been defined to date. The AR1 system is effective at pH 2.5 in minimal medium^14^ and is induced when *E. coli* cells are grown to stationary phase in complex medium buffered at pH 5.5 under oxidative conditions. Although AR1 induction was suppressed by glucose and requires the alternative sigma factor σ^S^ (RpoS) and cyclic AMP receptor protein (CRP)^15^, its precise regulatory mechanisms are unclear. The AR2–AR5 systems are composed of specific amino acid decarboxylases and antiporters which are induced at low pH. The amino acids required for AR2, AR3, AR4, and AR5 are glutamic acid, arginine, lysine, and ornithine, respectively^16,17^. The AR2 and AR3 systems primarily contribute to survival in extremely acidic environments (~pH 2.5), whereas AR4 and AR5 contribute to survival in moderately acidic environments (~pH 4.5)^16^. AR2 has been shown to be the most effective system for promoting survival under extreme acidic stress^15,18,19^.

The AR2 system is also known as the glutamic acid decarboxylase (GAD) system. It is composed of GadA and GadB, which are isozymes of pyridoxal-5′-phosphate (PLP)-dependent GADs, as well as the GadC antiporter. GadA and GadB catalyse the H^+^-consuming conversion of glutamic acid to γ-aminobutyric acid (GABA)^20,21^. GABA is released extracellularly by the GadC antiporter in exchange for the uptake of external glutamic acid^22,23^. The GAD system increases intracellular pH by consumption of cytoplasmic H^+^ and reversion of the electrical membrane potential, making the charge inside of the cell positive^24^. The positive internal charge seems to slow proton entry into the cell; the same strategy is present in multiple acidophiles that grow in extremely low pH environments^11,25^. The effectiveness of the GAD system for survival in the gastrointestinal tract has also been demonstrated *in vivo*, as the number of colonies isolated from calf faeces was significantly decreased relative to the wild-type strain when the GAD system-deficient *E. coli* strain was orally inoculated into calves^26^. A recent study suggests that genes encoding GAD constitute a significant proportion of human gut flora, which could affect human health via the gut-brain axis by producing GABA, a major inhibitory neurotransmitter of the mammalian central nervous system^27^.

Induction of the GAD system is controlled by an extraordinarily complex regulatory network. Nearly 20 proteins, including CRP, EvgA/S, GadE, GadX, GadW, H-NS, Lon, PhoP/Q, RNase E, σ^70^, σ^S^, SspA, TrmE, TopA, TorS/R, and YdeO, as well as 4 small non-coding RNAs (sRNAs), ArrS, DsrA, GadY, and GcvB, are known to regulate this system^16,28^. They form a complex, hierarchical regulatory cascade. Among them, a transcription factor, GadE, acts as a central activator of the GAD system. It directly induces *gadA*, *gadB*, and *gadC* transcription under moderately acidic conditions (~pH 5.5) and the stationary growth phase^29^, while other protein factors regulate *gadE* expression, directly or indirectly. Induction of the GAD system has been extensively investigated by genetic analyses^11,16^. However, the first event that initiates induction of the GAD system after exposure to acidic environments remains unknown. Elucidating this initial step would help us to better understand this network and is likely to be important in developing clinical preventive strategies against pathogenic *E. coli* infections.

Previously, we reported that the *E. coli tolC* mutant lacking the TolC outer membrane channel showed increased acid sensitivity, because GadA and GadB were not induced^30^. The TolC protein forms tripartite trans-periplasmic channels with inner membrane efflux pump proteins and their cognate periplasmic proteins^31,32^. This TolC-dependent efflux system contributes to exporting intracellular metabolites including porphyrin^33^, enterobactin^34^, and indole^35^, and multiple xenobiotics including antibiotics, dyes, organic solvents, and detergents^36–39^. Substrates of the TolC-dependent efflux system are thought to accumulate intracellularly when it does not function properly. Therefore, we hypothesised that a regulatory signal which represses expression of the GAD system would accumulate in *tolC*-mutant cells.

One of the substrates of the TolC-dependent efflux system, indole, is reported to suppress expression of GAD-related genes (*gadA*, *B*, *C*, *E*, and *X*) and reduce acid resistance in *E. coli*^40,41^. Indole is produced from tryptophan by the tryptophanase encoded by the *tnaA* gene. It has been reported that the production of indole is induced at high pH, probably owing to increased expression of TnaA, while it is repressed at low pH^42,43^. Although indole is a characteristic compound of *E. coli*^44^, high indole concentrations inhibit its growth^45^. Therefore, *E. coli* need to export indole; moreover, the TolC–AcrEF efflux pump can function as an indole exporter^35^. Based on these findings, we hypothesised that accumulated indole impairs GAD induction in *tolC*-mutant cells.

## Results and Discussion

### Effect of the *tnaA* mutation on the regulation of the GAD system in *tolC* mutant cells

**Figure 1:**
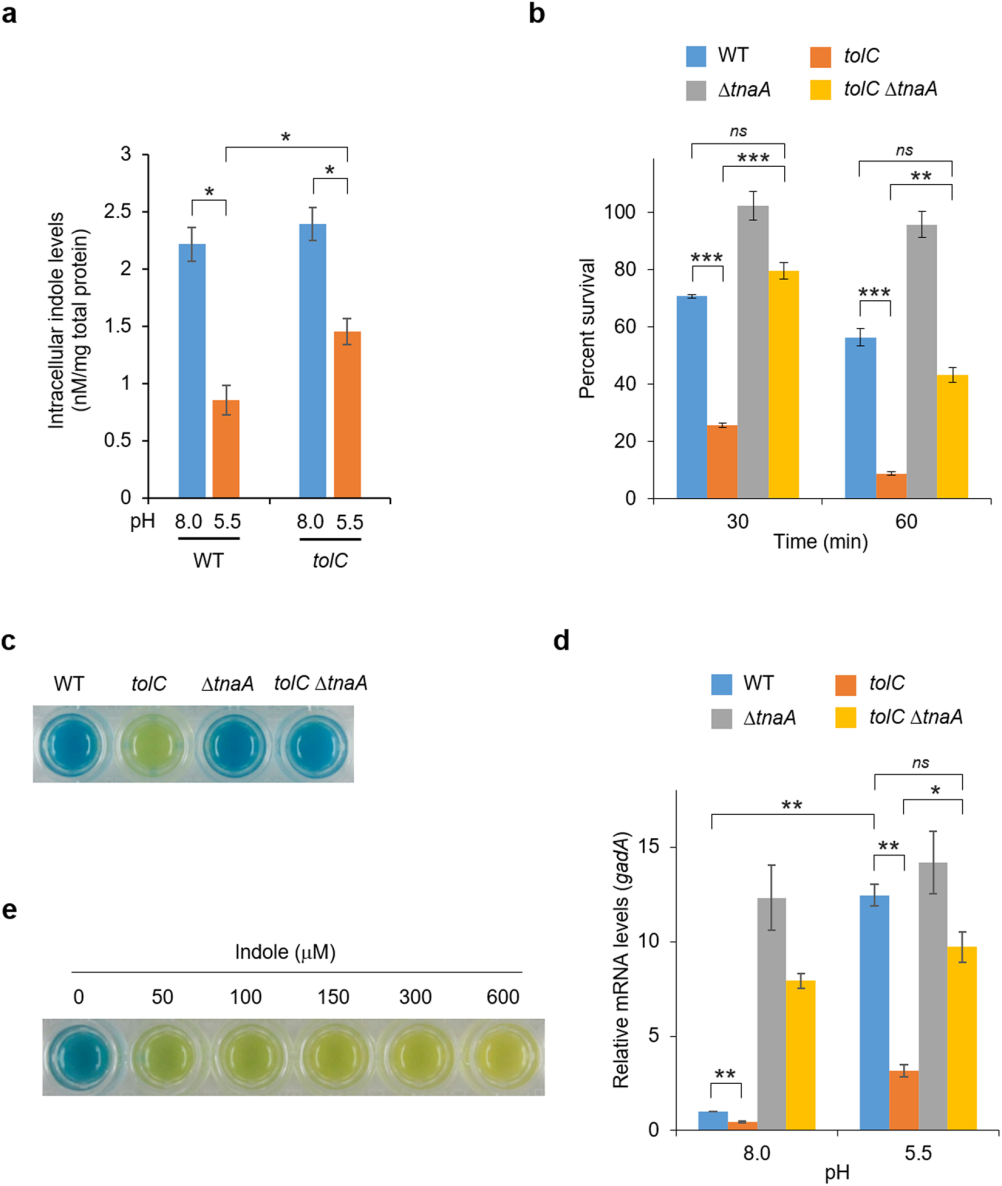
Effects of *tnaA* mutation and indole addition on acid tolerance and GAD expression. **(a)** Intracellular indole levels in wild-type MG1655 (WT) and its *tolC* derivative MG1655T (*tolC::*Tn10). Both strains were cultured for 30 min in LB at pH 8.0 or 5.5. Concentrations were normalized to total protein concentrations. (**b)** Percent survival of MG1655 (WT) and its derivatives, MG1655T (*tolC::*Tn10), TK20 (Δ*tnaA*), and TK12 (*tolC::*Tn*10* Δ*tnaA*) after acid challenge at pH 2.5. (**c)** Glutamic acid decarboxylase activity of *E. coli* MG1655 (WT) and its derivative mutants. Enzyme activity was qualitatively evaluated based on the colour change of the GAD reagent from yellow to blue. **(d)** Relative mRNA levels of *gadA* in *E. coli* MG1655 (WT) and derivative mutants grown at pH 8.0 or 5.5 quantified by qRT-PCR. The mRNA levels were normalised to those of 16S rRNA from the same RNA samples. (**e)** Glutamic acid decarboxylase activity of *E. coli* MG1655 grown in the presence of indole. Values are presented as mean ± SEM from three independent experiments (*n* = 3) and were statistically analysed using one-way ANOVA test with Holm’s post-hoc test in (**a**), and Bonferroni post-hoc test in (**b**,**d)** (*p < 0.05, **p < 0.01, ***p < 0.001). In (**c**,**e)**, photographs are representative of at least three independent experiments.

To test our hypothesis, we first measured the intracellular concentrations of indole in the wild-type and *tolC* mutant cells grown at pH 8.0 and 5.5. The *E. coli* wild-type strain MG1655 and its derivative, MG1655T (*tolC::*Tn10), were grown in Luria–Bertani (LB) broth buffered with 3-morpholinopropanesulfonic acid (MOPS) at pH 8.0 or 2-morpholinoethanesulfonic acid (MES) at pH 5.5 for 30 min. Intracellular indole concentrations were determined using Kovacs’ reagent. Intracellular indole levels in wild-type cells decreased when the pH was shifted to 5.5 (Fig. 1a). Decreased indole levels were also observed upon pH shift even in the *tolC* mutant cells, but the indole level at pH 5.5 in the *tolC* mutant cells was significantly higher than that in the wild-type (p < 0.05).

Then, we evaluated the effect of *tnaA* mutation on the expression of the GAD system in the *tolC* mutant. MG1655 and its derivatives, MG1655T (*tolC::*Tn10), TK20 (Δ*tnaA::kan*), and TK12 (*tolC::*Tn10 Δ*tnaA::kan*), were grown in LB broth buffered with MES (pH 5.5) for 16 h. Both were challenged at pH 2.5 in M9-glucose minimal medium supplemented with 1.5 mM l-glutamic acid. As reported previously^30^, the survival of the *tolC* mutant after acid challenge was markedly lower than that of wild-type *E. coli*. When the *tnaA* mutation was introduced into the *tolC* mutant, the survival rate was comparable to that of wild-type (Fig. 1b). Consistent with these results, the *tolC tnaA*-double mutant recovered the ability to induce GAD activity upon exposure to pH 5.5 (Fig. 1c). Total cellular RNA was extracted from *E. coli* cells cultured for 30 min in LB buffered with MOPS (pH 8.0) or MES (pH 5.5); *gadA*-mRNA levels were determined by quantitative real-time polymerase chain reaction (qRT-PCR). The *gadA* mRNA levels at pH 5.5 were 12-fold higher than those at pH 8.0 in wild-type cells. Although *gadA*-mRNA levels increased even in the *tolC* mutant after exposure to pH 5.5, they were clearly lower than those in wild-type. However, they were restored to levels comparable to wild-type cells by introducing the *tnaA* mutation into the *tolC* mutant (Fig. 1d). Under this condition, mRNA levels of the *gadE* gene, encoding the master regulator of the GAD system, were lower in the *tolC* mutant than those in the wild-type at pH 5.5; they were again restored by introducing the *tnaA* mutation into the *tolC* mutant (Supplementary Fig. S1). The intracellular indole concentrations were negligible in the *tnaA* and *tolC tnaA* mutant cells (data not shown). Considering these data, we hypothesised that higher indole levels in *tolC*-mutant cells at pH 5.5 suppressed induction of the GAD system. Consistent with this hypothesis, adding 50 – 600 μM indole to LB medium suppressed the induction of GAD activity in wild-type cells (Fig. 1e).

### RNase E-dependent degradation of *tnaA* mRNA upon acid treatment

**Figure 2:**
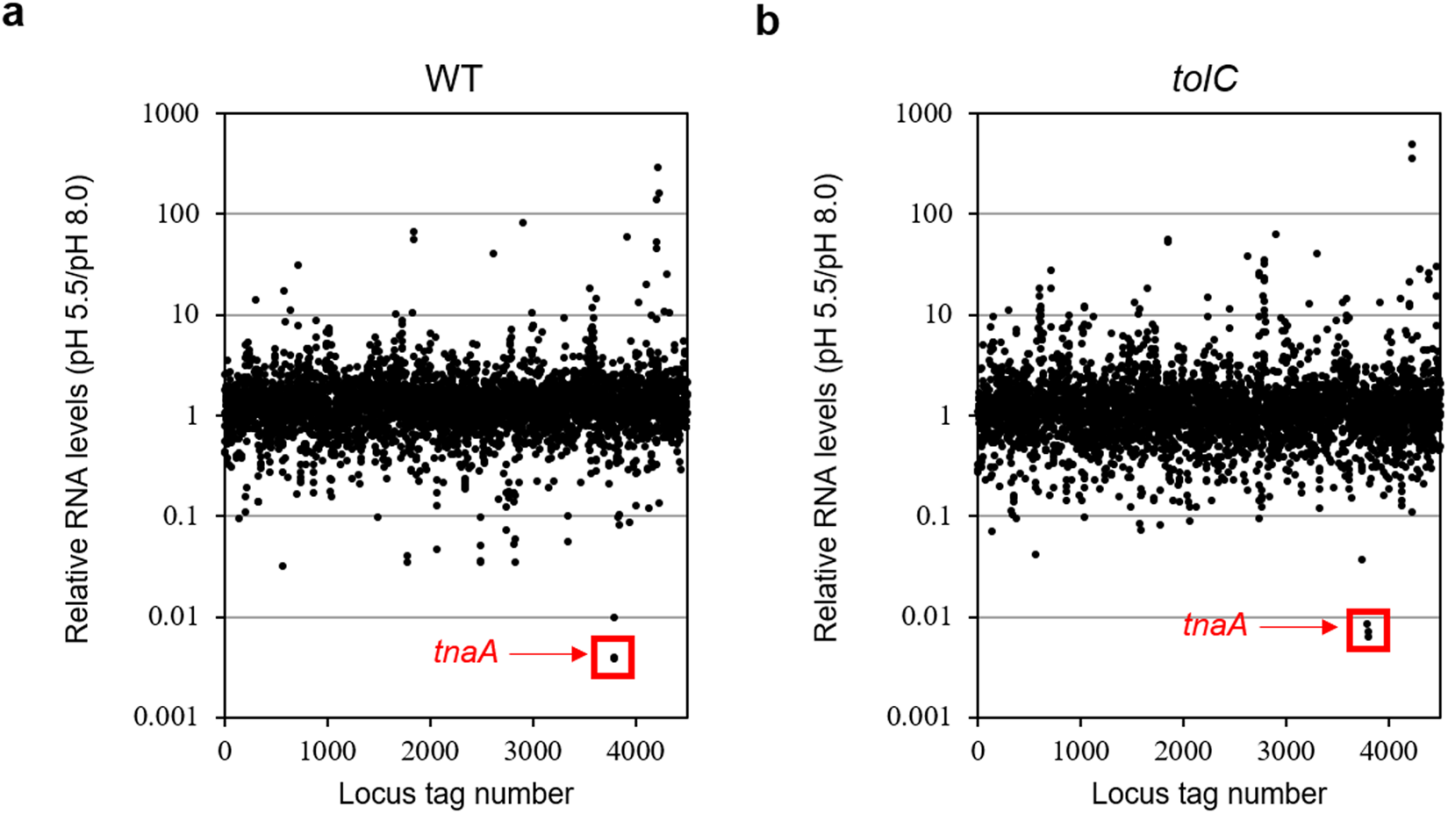
Gene-expression profiles of *E. coli* MG1655 and its *tolC* mutant upon acid treatment. **(a)** Relative RNA levels in *E. coli* MG1655 (WT) and **(b)** its *tolC::*Tn*10* mutant. Total cellular RNA was extracted from *E. coli* cells cultured at pH 8.0 or 5.5 for 30 min. Each dot represents the ratio of the RPKM value at pH 5.5 to that at pH 8.0 (*y* axis) for each gene, corresponding to the locus tag number (*x* axis). The *tnaA* gene is indicated with a red frame.

The decrease in intracellular indole levels upon pH shift to 5.5, even in the *tolC* mutant cells (Fig. 1a), suggested that indole synthesis was supressed in response to acidic conditions. To analyse the expression patterns of genes related to indole synthesis as well as the GAD system, we performed transcriptome analysis by RNA-sequencing (RNA-Seq) of MG1655 and its *tolC* mutant (MG1655T) cultured at pH 8.0 or pH 5.5 for 30 min (Fig. 2 and Table 1). As expected, the expression of GAD-related genes, including *gadA*, *gadB*, *gadC*, and *gadE*, was significantly increased in wild-type cells by acid treatment (Table 1 and Supplementary Table S1). Under these conditions, *tnaA* mRNA levels at pH 5.5 decreased drastically to <1% of those at pH 8.0, consistent with a previous transcriptome analysis^46^. These results suggest that the synthesis of tryptophanase, the only enzyme known to be responsible for indole production in *E. coli*, was shut down upon acid treatment.

**Table 1:** Gene-expression profiles of *E. coli* MG1655 and its *tolC* mutant upon acid treatment.

Gene	Product	pH 5.5/pH 8.0		*tolC*/WT	
		WT	*tolC*	pH 8.0	pH 5.5
*gadA*	Glutamate decarboxylase A	9.36	9.89	0.49	0.51
*gadB*	Glutamate decarboxylase B	2.13	2.76	0.56	0.73
*gadC*	Glutamate:GABA antiporter	2.27	2.75	0.58	0.70
*gadE*	GAD regulon activator	7.67	5.13	0.72	0.48
*tnaA*	Tryptophanase	0.0038	0.0064	0.25	0.42
*tnaB*	Tryptophan transporter	0.0040	0.0071	0.20	0.35
*tnaC*	Tryptophanase leader peptide	0.0099	0.0085	0.35	0.30
*nmpC*	Prophage porin	0.032	0.041	0.20	0.26
*cysJ*	Sulfite reductase, alpha subunit	0.034	0.37	0.66	7.00
*cysP*	Thiosulfate-binding protein	0.035	0.43	0.76	9.48
*ydjO*	Uncharacterized protein	0.035	0.08	1.06	2.49
*cysU*	ABC transporter	0.036	0.23	0.72	4.62
*ydjN*	Putative transporter	0.040	0.18	0.94	4.22
*yeeE*	Inner membrane protein	0.048	0.38	0.95	7.59

Representative genes showing altered expression levels in response to the pH shift to 5.5. Genes encoding core enzymes (*gadA*, *gadB*, and *gadC*) and the master regulator (*gadE*) of the GAD system are listed in the upper four rows of the table. Genes whose expression levels decreased significantly upon acidic shift are shown in ascending order of their RPKM ratios (pH 5.5/ 8.0) in the wild-type in the lower ten rows. The two central columns show the RPKM ratios (pH 5.5/ 8.0) of MG1655 (WT) and its *tolC* mutant. The two right columns show the ratios of RPKM values of *tolC* mutant to that of MG1655 at pH 8.0 or 5.5. Mean of RPKM ratios was calculated from two independent experiments.

In the *tolC*-mutant, *gad*-expression levels were approximately half of wild-type levels at pH 5.5 and 8.0 (Table 1). These results suggest that expression of the *gad* genes was constantly repressed in the cells of the *tolC* mutant, independent from extracellular pH. The drastic decrease in *tnaA*-mRNA levels also occurred in the cells of the *tolC* mutant, suggesting that it occurred prior to a decrease in intracellular indole levels.

**Figure 3:**
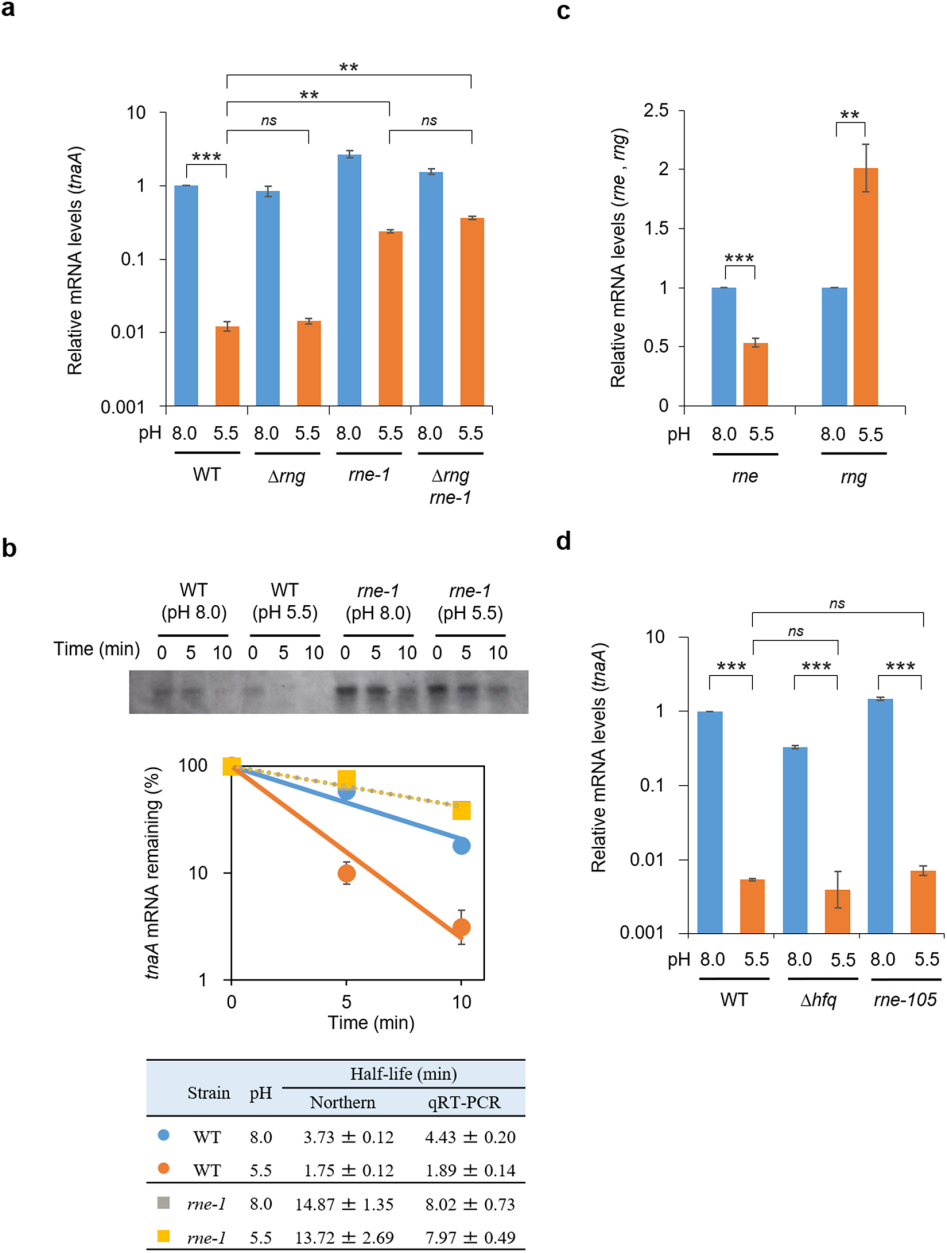
pH-responsible degradation of *tnaA* mRNA by RNase E. **(a)** Relative mRNA levels of *tnaA* in *E. coli* MG1655 (WT) and its RNase mutants at pH 8.0 or 5.5 quantified by qRT-PCR. Total cellular RNAs were extracted from *E. coli* cells cultured at 42 °C for 30 min in LB at pH 8.0 or 5.5. The *rne-1* mutant produces a temperature-sensitive variant of RNase E. **(b)** Relative *tnaA* mRNA levels remaining after rifampicin treatment at pH 8.0 or 5.5 in MG1655 and its *rne-1* mutant. Top, a representative northern blot. A part of the blot is shown. A whole image of the blot is shown in Supplementary Fig. S2. Middle, relative *tnaA*-mRNA levels quantified using qRT-PCR. Bottom, half-lives of *tnaA* mRNA calculated using both the methods. **(c)** Relative mRNA levels of *rne* and *rng*, encoding RNase E and RNase G, respectively, quantified by qRT-PCR at pH 8.0 or 5.5. **(d)** Relative *tnaA* mRNA levels in MG1655 (WT) and its derivatives, the *rne-105* mutant and Δ*hfq* mutant, at pH 8.0 or 5.5 quantified by qRT-PCR. The *rne-105* mutant produces an RNase E variant lacking the C-terminal half, which is known to act as a scaffold for degradosome formation. The mRNA levels of the target genes were normalised to those of 16S rRNA from the same RNA samples. Values are presented as means ± SEM from three independent experiments and were statistically analysed using one-way ANOVA with Bonferroni post-hoc test in (**a**,**b**,**d**), or using the two-tailed Student’s t-test in **c** (**p < 0.01, ***p < 0.001).

Since the decrease in the *tnaA*-mRNA levels occurred so quickly upon acid treatment, almost within a single doubling time, we hypothesised that a specific RNase may be involved. We therefore examined the effects of mutations of RNase E and RNase G, major endo-type RNases that initiate mRNA decay in *E. coli*^47,48^ and are encoded by the *rne* and *rng* genes, respectively. As shown in Fig. 3a, *tnaA* mRNA levels decreased ~100-fold upon acid treatment in wild-type cells, consistent with the RNA-Seq data (Fig. 2 and Table 1). After introducing the temperature-sensitive *rne-1* mutation, the decrease in *tnaA*-mRNA levels was significantly attenuated. In contrast, the *rng::cat* mutant, which does not produce RNase G, showed a negligible effect. These results suggest that initiation of *tnaA*-mRNA degradation under acidic conditions is primarily catalysed by RNase E.

We then performed a rifampicin-chase experiment to measure the turnover rate of *tnaA* mRNA under acidic conditions. Exponentially growing cells at pH 8.0 or 5.5 were treated with 150 μg/ml rifampicin to prevent further initiation of transcription. Total cellular RNA was isolated at multiple times after adding rifampicin. Rates of *tnaA*-mRNA decay were determined by northern blotting (Fig. 3b). We found that decay of full-length *tnaA* mRNA was accelerated upon a shift to pH 5.5 in the wild-type control. In contrast, in the *rne-1* mutant *tnaA* mRNA decay was slower at both pH 8.0 and 5.5. Acceleration of *tnaA* mRNA decay upon pH shift was completely abrogated in the *rne-1* mutant. The half-lives of full-length *tnaA* mRNA were estimated from band intensities; they were 3.73 min at pH 8.0 and 1.75 min at pH 5.5 in the wild-type, and 14.87 min at pH 8.0 and 13.72 min at pH 5.5 in the *rne-1* mutant. The half-lives of *tnaA* mRNA, estimated using qRT-PCR, were consistent with the northern blot data; 4.43 min at pH 8.0 and 1.89 min at pH 5.5 in the wild-type, and 8.02 min at pH 8.0 and 7.97 min at pH 5.5 in the *rne-1* mutant. These results suggest that the mRNA degradation rate for *tnaA* increased after the acidic shift in an RNase E-dependent manner. Considering that indole repressed GAD expression, this acid-responsive mRNA degradation may trigger induction of the GAD system in response to an acidic environment.

To investigate the mechanism of acid-responsive degradation of *tnaA* mRNA, we determined whether degradation was caused by increased RNase E production. Total cellular RNA was extracted from wild-type *E. coli* under the conditions used for RNA-Seq and the mRNA levels of *rne* and *rng* were determined by qRT-PCR. When the pH was shifted to 5.5, *rne* mRNA levels in wild-type cells did not increase (Fig. 3c). The RNA-seq data also indicated that the relative activity of RNase E did not change upon acid treatment, since the levels of other known RNase E substrates were unchanged (Supplementary Table S2). In addition, we found that mRNA levels of the known transcriptional regulators of *tnaA* did not change upon acid shift, suggesting that the acid-induced decrease in *tnaA* mRNA abundance was independent of transcription (Supplementary Table S3).

It has been reported that sRNA and the RNA chaperone Hfq often participate in mRNA degradation initiated by RNase E^49–52^. In addition, the C-terminal half of RNase E is known to act as a scaffold for the formation of degradosome complexes which degrade RNA^47,53^. We therefore examined whether the sRNA/Hfq and/or the degradosome were involved in *tnaA*-mRNA degradation under acidic conditions. Total cellular RNA was isolated from wild-type (MG1655), Δ*hfq* mutant (TK50), and *rne-*10*5* mutant (TK60, which produces RNase E lacking a C-terminal half) *E. coli*, after culture for 30 min at pH 8.0 or 5.5. The amounts of *tnaA* mRNA were measured by qRT-PCR. In wild-type cells, *tnaA* mRNA expression was decreased by more than 100-fold by the acidic shift. Under this condition, *tnaA* mRNA expression in both the Δ*hfq* and *rne-*10*5* mutants decreased to almost the same level as that of wild-type cells (Fig. 3d). These data indicate that *tnaA* mRNA degradation initiated by RNase E in acidic conditions was independent of RNA-degradation cofactors, including sRNA/Hfq and the degradosome.

Acid-induced degradation was limited to *tnaA* mRNA among RNase E substrates (Fig. 2, Table 1, and Supplementary Table S2), suggesting that neither expression levels nor the activity of RNase E had changed. Moreover, this event was independent of both sRNA/Hfq and RNA degradosomes. Considering these findings, the behaviour of the substrate itself — for example, the secondary structure of the *tnaA* mRNA — may change upon acid treatment. Recently, a pH-responsive riboregulator, which forms two different secondary structures in response to extracellular pH and changes gene expression, was reported in *E. coli*^54^. It is possible that *tnaA* mRNA forms an alternative secondary structure under acidic conditions, which may allow RNase E to cleave it. To understand the mechanism underlying acid-induced degradation of *tnaA* mRNA, further analysis is required, including *in vitro* assays and mapping of cleavage site(s) of the *tnaA* mRNA by RNase E under neutral and acidic conditions.

**Figure 4:**
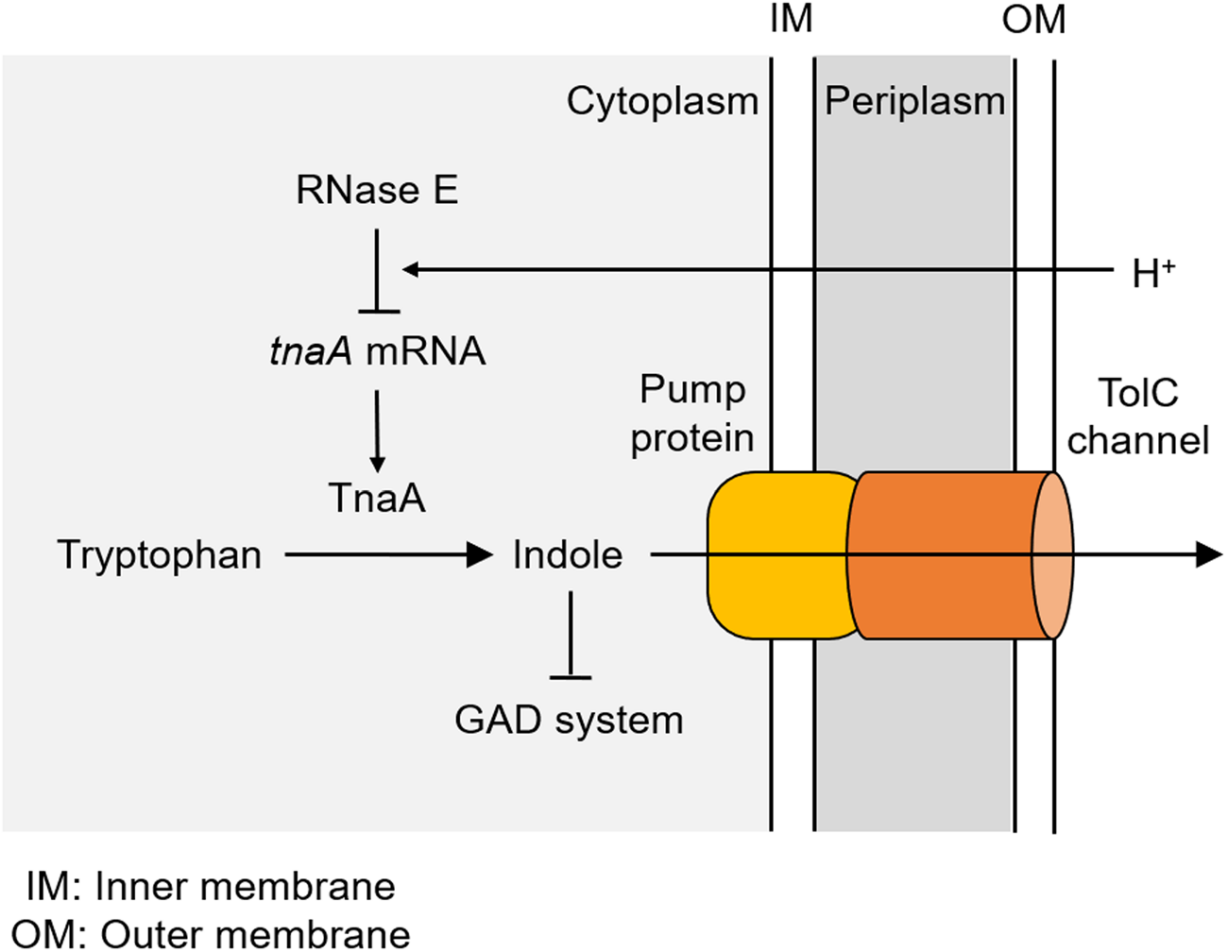
Model for GAD induction. Expression of the GAD system is repressed in the presence of indole under neutral and alkaline conditions. When cells are exposed to an acidic environment, tryptophanase synthesis is immediately shut down by RNase E-dependent degradation of *tnaA* mRNA. The remaining indole is excreted by the TolC-dependent efflux pump. Consequently, the intracellular concentration of indole decreases, triggering induction of the GAD expression.

Based on the results presented above, we propose the following model to explain the induction of GAD in *E. coli* (Fig. 4): (1) expression of the GAD system is repressed in the presence of indole under neutral and alkaline conditions; (2) tryptophanase synthesis is immediately shut down by RNase E-dependent degradation of *tnaA* mRNA when cells in an acidic environment; (3) the remaining indole is excreted by the TolC-dependent efflux pump, and (4) consequently, intracellular levels of indole decrease, which triggers induction of the GAD system.

The data generated in this study demonstrate that deletion of the *tnaA* gene restored expression of the GAD system in the *tolC* mutant and that indole addition suppressed GAD induction in wild-type cells (Fig. 1). These results suggest that indole signalling negatively regulates GAD expression in *E. coli*. Indole, which is widespread in animal digestive tracts owing to its production by gut bacteria, has received considerable attention as it plays important roles in bacterial pathogenesis and human immunity as an intercellular and interkingdom signalling molecule^55,56^. Recently, increasing evidence has indicated a variety of functions for indole in bacterial physiology, including biofilm formation, motility, virulence, plasmid stability, and antibiotic resistance^40,41,45,57–59^. However, the precise mechanisms by which indole influences these diverse phenomena have not yet been clarified^60^.

Our RNA-seq data revealed several regulators of the GAD system for which RNA levels decreased in the *tolC* mutant, relative to the wild-type, at pH 5.5, probably due to the accumulated indole (Supplementary Table S1). The RNA levels of *gadX*, *gadY*, *arrS*, *ydeO*, and *evgA* were as low as or even lower than those of *gadE*, which encodes the master regulator of the GAD system. Analysis of interactions between indole and these regulators may help to reveal the mechanisms involved in its regulation of gene expression. Since extracellular indole can repress expression of the GAD system, agonistic substances which mimic indole signalling may be viable candidates for anti-infectious drugs against pathogenic *E. coli*.

## Methods

### Bacterial strains and growth media

The *E. coli* wild-type K-12 strain MG1655 and its derivatives were used in this study. Mutant strains were constructed by P1 phage-mediated transduction using MG1655 as an acceptor strain and strains carrying the appropriate mutations as donor strains (Supplementary Table S4). *Escherichia coli* were grown in LB broth (1% polypeptone, 0.5% yeast extract, and 1% NaCl, pH 7.0). When necessary, the pH was adjusted by adding 0.5 ml of either 1 M MOPS buffer (pH 8.0) or 1 M MES buffer (pH 5.5) per 4.5 ml of LB broth. For the acid-resistance assays, we used M9-glucose minimal medium (6.8 g/l Na_2_HPO_4_, 3.0 g/l KH_2_PO_4_, 0.5 g/l NaCl, 1.0 g/l NH_4_Cl, 2 mM MgSO_4_, 0.1 mM CaCl_2_, and 4 g/l glucose) supplemented with 1.5 mM l-glutamic acid and adjusted to pH 2.5 with HCl.

### Indole assay

*E. coli* were aerobically grown at 37 °C in 4.5 ml of LB broth (pH 7.0). When the OD_660_ reached ~0.5, 0.5 ml of 1 M MOPS (pH 8.0) or 1 M MES (pH 5.5) was added to the culture. After 30 min of incubation, cells were harvested by centrifugation, quickly rinsed, and resuspended in 0.25 ml of 50 mM sodium phosphate buffer (pH 7.0). Cells were then sonicated with a Bioruptor UCD-250 (Cosmo Bio Co., Ltd., Tokyo, Japan). A 0.2-ml sample was mixed immediately with 0.2 ml of Kovacs’ indole reagent (Merck KGaA, Darmstadt, Germany) and incubated for 2 min at room temperature. The reaction mixture was centrifuged at 15,800 × *g* for 5 min and the upper layer was collected. The concentrations of indole were determined spectrophotometrically by measuring absorbance at 540 nm. To obtain intracellular indole concentrations, these measurements were normalized to total protein concentrations. Values are presented as means ± SEM from three independent experiments and were statistically analysed using the one-way ANOVA with Holm’s post-hoc test (*p < 0.05).

### Acid-resistance assay

*E. coli* grown in 5 ml of LB broth buffered with MES (pH 5.5) at 37 °C for 16 h were challenged with acid by diluting the cells 1000-fold into 5 ml of M9-glucose minimal medium supplemented with 1.5 mM l-glutamic acid (pH 2.5). After acid exposure for 30 or 60 min at 37 °C, the cells were plated on LB plates (1.5% agar) after appropriate dilution. The plates were incubated at 37 °C overnight and percent survival was calculated by counting colony-forming units (CFUs) after acid challenge, relative to those of unexposed control cells. After acid exposure, the pH of the culture was checked to ensure that it was within 0.2 pH units of that of the original uninoculated medium. Survival values are presented as means ± SEM from three independent experiments and were statistically analysed using one-way ANOVA with the Bonferroni post-hoc test (**p < 0.01, ***p < 0.001).

### Glutamic acid decarboxylase assay

Activity was assessed using GAD reagent (1 g/l l-glutamic acid, 0.05 g/l bromocresol green, 90 g/l NaCl, and 3 ml/l Triton X-100) as described previously^52^ with minor modifications. *E. coli* were aerobically grown at 37 °C in 4.5 ml of LB broth (pH 7.0). When the OD_660_ reached ~0.5, 0.5 ml of 1 M MES (pH 5.5) was added and growth was continued for 30 min. Cells were harvested by centrifugation and washed with 1 ml of 0.85% NaCl to adjust cell concentrations. An aliquot of each cell suspension (6.0 × 10^8^ cells) was transferred to 200 μl of GAD reagent. The reaction mixtures were incubated at 35 °C for 6 h and then evaluated qualitatively for decarboxylase activity, based on a colour change from yellow to blue. To examine the effects of indole addition on GAD expression, *E. coli* cells were grown in the absence or presence of the indicated concentrations of indole (50–600 μM) in 4.5 mL of LB broth (pH 7.0), then GAD activities were evaluated as described above.

### Total RNA extraction

*E. coli* were aerobically grown in LB broth at 37 °C. When the OD_660_ reached ~0.5, 0.5 ml of 1 M MOPS (pH 8.0) or MES (pH 5.5) was added to the cultures. In the case of *rne-1*-mutant strains, cells were grown in LB broth at 30 °C, and the growth temperature was shifted to 42 °C just before adding MOPS (pH 8.0) or MES (pH 5.5). After a 30-min incubation, the cells were harvested by centrifugation and suspended in RNA Protect Bacterial Reagent (Qiagen, Venlo, Netherlands) to stabilize cellular RNA. Total cellular RNA was isolated with the RNeasy Mini Kit (Qiagen) according to the manufacturer’s instructions. RNA was treated with DNase I at room temperature for 1 h. In the rifampicin-chase experiments, cells were treated with 150 μg/ml rifampicin immediately after adding MOPS or MES buffer, then total cellular RNA was isolated 0, 5, or 10 min after rifampicin addition.

### RNA-Seq

One microgram of total RNA was used to create complementary DNA (cDNA) libraries. Ribosomal RNA was removed using the RiboZero Bacteria Kit (Illumina, San Diego, CA, USA), per the manufacturer’s protocol. Sequencing libraries were prepared using the NEBNext mRNA Library Prep Kit for Illumina (New England Biolabs, Inc., Ipswich, MA, USA), with the following modifications. Random hexamer primers were used for reverse transcription. After second-strand synthesis, the double-stranded cDNA was fragmented to an average length of 300 base pairs using a Covaris S2 sonication system (Covaris, Woburn, CA, USA). One hundred cycles of paired-end sequencing were carried out using the HiSeq. 2500 system, according to the manufacturer’s specifications (Illumina). After the sequencing reactions were complete, the Illumina analysis pipeline (CASAVA 1.8.0) was used to process the raw sequencing data. The reads were trimmed using CLC Genomics Workbench ver. 10.0.1. with the following parameters: Phred quality score >30; removing the terminal 15 nucleotides from the 5′ end and two nucleotides from the 3′ end; and removing truncated reads of <30 nucleotides. Trimmed reads were mapped to all genes in *E. coli* str. K-12 substr. MG1655 (accession number: NC_000913.3) using CLC Genomics Workbench version 10.0.1. (Qiagen) with the following parameters: length fraction: 0.7; similarity fraction: 0.9; maximum number of hits for a read: 1. The RNA level for each gene was calculated by counting the number of reads mapped to each gene, normalized by calculating reads per kilobase of transcript per million mapped reads (RPKM) values, and shown as mean from two independent experiments. Original sequence reads have been deposited in the DRA/SRA database under accession numbers DRR162758–DRR162761.

### Northern blotting

Total RNA (6.8 μg) was separated by electrophoresis using a Formaldehyde-Free RNA Gel Kit (1.2% agarose) (Amresco, Solon, OH, USA). The gels were blotted onto positively charged nylon membranes (Roche Diagnostics, Mannheim, Germany) using the capillary transfer method. A digoxigenin (DIG)-labelled antisense DNA probe targeting the entire coding region of *tnaA* mRNA was produced using the PCR DIG Probe Synthesis Kit (Roche Diagnostics). To generate the probe, specific DNA fragments were amplified using PCR with the primers 5′-AAACATCTCCCTGAACCGTTCCGCATTC-3′ and 5′-TCGACCAGATACTGTACCTGCGCGATAC-3′.

Prehybridization (30 min) and hybridization (16 h) were performed in DIG Easy Hyb (Roche Diagnostics) at 50 °C. The membrane was washed using DIG Wash and Block Buffer Set (Roche Diagnostics) according to the manufacturer’s instructions. Hybridization was detected using the CDP-Star reagent (Roche Diagnostics) with an exposure time of 5 min. The amounts of *tnaA* mRNA were measured on the basis of the band intensities using ImageJ software.

### Quantitative RT-PCR

The mRNA levels were quantified by qRT-PCR using an Eco Real-Time PCR System (Illumina) and the QuantiFast SYBR Green RT-PCR Kit (Qiagen). Twenty nanograms of each total RNA sample were used for qRT-PCR with each primer pair. Each target-gene mRNA level was normalised to a reference gene transcript (16S rRNA) from the same RNA sample. The cycle threshold for each sample was generated according to the procedures described in the Eco Real-Time PCR System User Guide. The sequences of the primers used are shown in Supplementary Table S5. Relative mRNA expression levels are presented as means ± SEM from three independent experiments and analysed using the two-tailed Student’s t-test or one-way ANOVA with the Bonferroni post-hoc test (*p < 0.05, **p < 0.01, ***p < 0.001).

## Supplementary information

Supplementary Information.

## Data Availability

All data generated or analysed during this study are included in this article and its supplementary information file. Original RNA-Seq reads have been deposited in the DRA/SRA database under accession numbers DRR162758-DRR162761.
