# Supplementary material for: RNase E-dependent degradation of tnaA mRNA encoding tryptophanase is prerequisite for the induction of acid resistance in Escherichia coli
Source: Sci Rep. 2020 Apr 28;10:7128. doi: 10.1038/s41598-020-63981-x (PMC7188888; doi:10.1038/s41598-020-63981-x)
Supplement: Supplementary file 1 — Supplementary Information. [file 41598_2020_63981_MOESM1_ESM.pdf]

RNase E-dependent degradation of *tnaA* mRNA encoding tryptophanase is prerequisite for the induction of acid resistance in *Escherichia coli*

Takeshi Kanda[1], Genta Abiko[1], Yu Kanesaki[2], Hirofumi Yoshikawa[3], Noritaka Iwai[1], Masaaki Wachi[1,*]

[1]Department of Life Science and Technology, Tokyo Institute of Technology, 4259 Nagatsuta, Midori-ku, Yokohama 226-8501, Japan

[2]NODAI Genome Research Center, Tokyo University of Agriculture, 1-1-1 Sakuragaoka, Setagaya-ku, Tokyo 156-8502, Japan

(Present address: Research Institute of Green Science and Technology, Shizuoka University, 836 Ohya, Suruga-ku, Shizuoka 422-8529, Japan)

[3]Department of Bioscience, Tokyo University of Agriculture, 1-1-1 Sakuragaoka, Setagaya-ku, Tokyo 156-8502, Japan

*Corresponding author

Email: mwachi@bio.titech.ac.jp

Tel: +81-45-924-5770

Fax: +81-45-924-5820

Supplementary Figure S1: Relative mRNA levels of *gadE* in MG1655 and its derivative mutants at pH 8.0 or 5.5

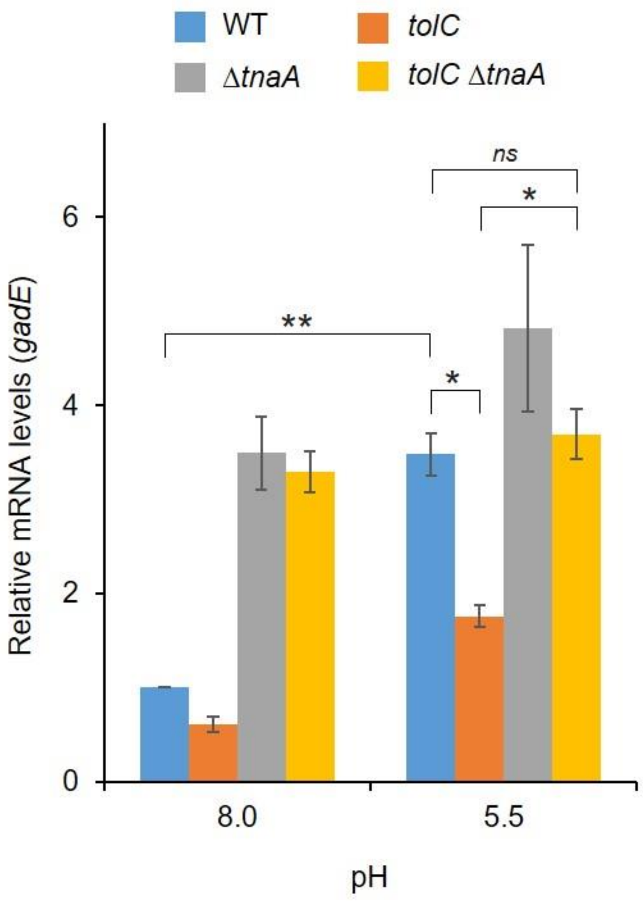

Relative mRNA levels of *gadE* in *E. coli* MG1655 (WT) and its derivative mutants grown at pH 8.0 or 5.5 were quantified using qRT-PCR. The *gadE*-mRNA levels were normalised to the reference gene transcript (16S rRNA) from the same RNA samples. Values are presented as means ± SEM from three independent experiments ($n = 3$) and were analysed using one-way ANOVA with Bonferroni post-hoc test (*p < 0.05, **p < 0.01).

Supplementary Figure S2: A whole image of the northern blot of Fig. 3b.

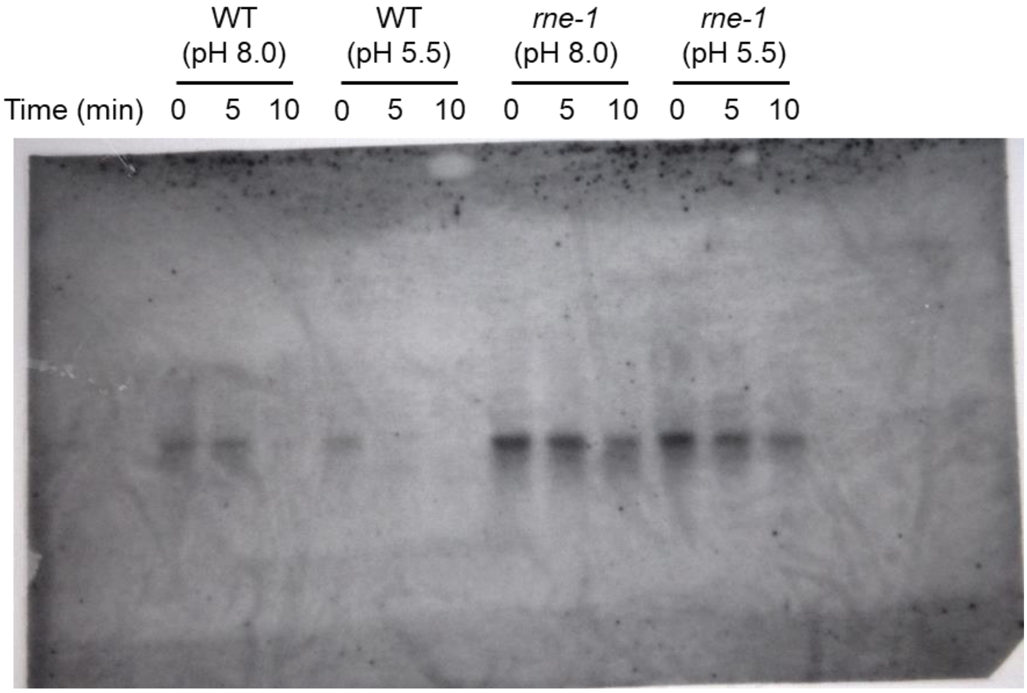

The photo was taken by a CCD camera and the image was not processed by any image processing softwares.

Supplementary Table S1: RPKM ratios of genes encoding core enzymes and regulators of the GAD system in MG1655 and its *tolC* mutant cells at pH 8.0 or 5.5.

Gene	Product	pH 5.5/pH 8.0		*tolC*/WT	
		WT	*tolC*	pH 8.0	pH 5.5
gadW	Transcriptional activator of *gadA* and *gadBC*, and repressor of *gadX*	11.76	8.70	0.75	0.55
gadA	Glutamate decarboxylase A	9.36	9.89	0.49	0.51
gadE	GAD regulon activator	7.67	5.13	0.72	0.48
gadX	Acid resistance regulon transcriptional activator	6.75	2.89	0.97	0.41
gadY	Antisense sRNA, which positively regulates *gadX* and *gadW*	6.67	3.14	1.01	0.47
dsrA	Antisense sRNA, which enhances translation of *rpoS*	6.55	3.53	1.96	1.05
arrS	Antisense sRNA, which enhances transcription of *gadE*	6.02	4.16	0.68	0.47
gadC	Glutamate:gamma-aminobutyric acid antiporter	2.27	2.75	0.58	0.70
gadB	Glutamate decarboxylase B	2.13	2.76	0.56	0.73
torR	Response regulator in two-component regulatory system with TorS	2.07	2.30	0.93	1.03
phoP	Response regulator in two-component regulatory system with PhoQ	2.02	1.89	1.02	0.95
phoQ	Sensory histidine kinase in two-component regulatory system with PhoP	1.79	1.56	1.03	0.90
ydeO	UV-inducible global regulator	1.78	0.96	0.86	0.46
evgS	Hybrid sensory histidine kinase in two-component regulatory system with EvgA	1.63	0.91	1.09	0.61
crp	cAMP-activated global transcription factor, which mediates catabolite repression	1.63	1.62	0.69	0.68
evgA	Response regulator in two-component regulatory system with EvgS	1.36	0.76	0.87	0.48
torS	Hybrid sensory histidine kinase in two-component regulatory system with TorR	1.35	1.02	0.96	0.72
hns	Global DNA-binding transcriptional dual regulator	1.09	0.95	1.04	0.91
lon	DNA-binding ATP-dependent protease	1.04	1.05	0.77	0.78
sspA	Stringent starvation protein A	1.03	1.08	0.95	0.98
mnmE	tRNA U34 5-methylaminomethyl-2-thiouridine modification GTPase	1.02	0.93	1.01	0.92
rpoD	Sigma D (sigma 70) factor	1.01	1.50	0.95	1.42
gcvB	Antisense sRNA, which represses *oppA*, *dppA*, *gltI* and *livJ*	0.99	0.48	1.36	0.65
rpoS	Sigma S (sigma 38) factor	0.90	1.15	0.95	1.20
topA	DNA topoisomerase I	0.74	0.68	0.98	0.90
rne	Endoribonuclease	0.72	0.84	0.83	0.97

Fold change

0.5 2 4 8

Supplementary Table S2: RPKM ratios for representative RNA substrates of RNase E upon acid treatment in MG1655 cells.

Gene	Product	pH 5.5/pH 8.0 (WT)	Reference
dnaG	DNA primase	0.86	1
ompA	Outer membrane protein A	1.14	2
ptsG	Glucose-specific PTS enzyme, IIB component	1.63	3
rne	Endoribonuclease E	0.72	4
rpsO	30S ribosomal subunit protein S15	0.93	5
rpsT	30S ribosomal subunit protein S20	0.84	6
ryhB	sRNA antisense regulator mediating positive Fur regulon response	1.29	2

PTS, phosphoenolpyruvate: carbohydrate phosphotransferase system

Supplementary Table S3: RPKM ratios of genes encoding *tna*-operon regulators upon acid treatment in MG1655 and its *tolC* mutant cells.

Gene	Product	pH 5.5/pH 8.0		Reference
		WT	*tolC*	
torS	Hybrid sensory histidine kinase in two-component regulatory system with TorR	1.35	1.02	7
torR	Response regulator in two-component regulatory system with TorS	2.07	2.30	7
torI	Response regulator inhibitor for *tor* operon	1.07	0.45	8
crp	cAMP-activated global transcription factor, mediator of catabolite repression	1.63	1.62	9
cyaA	Adenylate cyclase	1.20	1.51	10

Supplementary Table S4: Bacterial strains used in this study.

Strain	Relevant genetic marker(s)	Donor (Reference)	Source or reference
MG1655	Wild type		Laboratory stock
MG1655T	MG1655 *tolC*::Tn*10*		11
TK20	MG1655 Δ*tnaA*::*kan*	JW3686 (Keio collection[12])	This study
TK12	MG1655T Δ*tnaA*::*kan*	JW3686 (Keio collection[12])	This study
TK30	MG1655 *rng*::*cat*	GW11[13]	This study
TK40	MG1655 *rne-1*	GW20[13]	This study
TK34	TK30 *rne-1*	GW20[13]	This study
TK50	MG1655 *hfq10*::*cat*	HAT10[14]	This study
TK60	MG1655 *rne-105* (*smb-105*)	BZ5[15]	This study

Mutant strains were constructed by P1 phage-mediated transduction using MG1655 or its derivatives as acceptors and strains carrying the appropriate mutations as donors.

Supplementary Table S5: DNA primers used for qRT-PCR analysis.

Target gene	5′ primer sequence (5′→3′)	3′ primer sequence (5′→3′)	Reference
16S rRNA	GTTAATACCTTTGCTCATTGA	ACCAGGGTATCTAATCCTGTT	16
gadA	TTACCAGGTTGCCGCTTATC	ACGCAGACGTTCAGAGAGGT	17
tnaA	CTTTAAACATCTCCCTGAACCGTTC	GTGCCGCTGTCGGTCAGTAAATCG	This study
gadE	CTTTTCTTTTACAGGGCTTTTGGCAG	CGCTTCTTCATCAAGGATATGATTG	This study
rne	GAATGTTAATCAACGCAACTCAGC	GGTTCAATGCGGGTGATTTTAC	This study
rng	CTGAATTGTTAGTAAACGTAACGCC	AAGTACACGACTTACACGACCCTTG	This study

Supplementary references

1. Yajnik, V. & Godson, G. N. Selective decay of *Escherichia coli dnaG* messenger RNA is initiated by RNase E. *J. Biol. Chem.* **268,** 13253–13260 (1993).

2. Moll, I., Afonyushkin, T., Vytvytska, O., Kaberdin, V. R. & Bläsi, U. Coincident Hfq binding and RNase E cleavage sites on mRNA and small regulatory RNAs. *RNA* **9,** 1308–1314 (2003).

3. Kimata, K., Tanaka, Y., Inada, T. & Aiba, H. Expression of the glucose transporter gene, *ptsG*, is regulated at the mRNA degradation step in response to glycolytic flux in *Escherichia coli*. *EMBO J.* **20,** 3587–3595 (2001).

4. Jain, C. & Belasco, J. G. RNase E autoregulates its synthesis by controlling the degradation rate of its own mRNA in *Escherichia coli*: unusual sensitivity of the *rne* transcript to RNase E activity. *Genes Dev.* **9,** 84–96 (1995).

5. Braun, F., Hajnsdorf, E. & Régnier, P. Polynucleotide phosphorylase is required for the rapid degradation of the RNase E-processed *rpsO* mRNA of *Escherichia coli* devoid of its 3' hairpin. *Mol. Microbiol.* **19,** 997–1005 (1996).

6. Coburn, G. A. & Mackie, G. A. Reconstitution of the degradation of the mRNA for ribosomal protein S20 with purified enzymes. *J. Mol. Biol.* **279,** 1061–1074 (1998).

7. Bordi, C., Théraulaz, L., Méjean, V. & Jourlin-Castelli, C. Anticipating an alkaline stress through the Tor phosphorelay system in *Escherichia coli*. *Mol. Microbiol.* **1,** 211–223 (2003).

8. Ansaldi, M., Théraulaz, L., & Méjean, V. TorI, a response regulator inhibitor of phage origin in *Escherichia coli*. *Proc. Natl. Acad. Sci.* **25,** 9423–9428 (2004).

9. Deeley, M. C. & Yanofsky, C. Transcription initiation at the tryptophanase promoter of *Escherichia coli* K-12. *J. Bacteriol.* **2,** 942–951 (1982).

10. Görke, B. & Stülke, J. Carbon catabolite repression in bacteria: many ways to make the most out of nutrients. *Nat. Rev. Microbiol.* **8,** 613–624 (2008).

11. Deininger, K. N. W. *et al*. A requirement of TolC and MDR efflux pumps for acid adaptation and GadAB induction in *Escherichia coli*. *PLoS One* **6,** e18960 (2011).

12. Baba, T. *et al*. Construction of *Escherichia coli* K-12 in-frame, single-gene knockout mutants: the Keio collection. *Mol. Syst. Biol.* **2,** 2006.0008 (2006).

13. Wachi, M., Umitsuki, G. & Nagai, K. Functional relationship between *Escherichia coli* RNase E and the CafA protein. *Mol. Gen. Genet.* **253,** 515–519 (1997).

14. Wachi, M., Takada, A. & Nagai, K. Overproduction of the outer-membrane proteins FepA and FhuE responsible for iron transport in *Escherichia coli hfq::cat*

mutant. *Biochem. Biophys. Res. Commun.* **264,** 525–529 (1999).

15. Kido, M. *et al*. RNase E polypeptides lacking a carboxyl-terminal half suppress a *mukB* mutation in *Escherichia coli*. *J. Bacteriol.* **178,** 3917–3925 (1996).

16. Gao, W., Zhang, W. & Meldrum, D. R. RT-qPCR based quantitative analysis of gene expression in single bacterial cells. *J. Microbiol. Methods* **85,** 221–227 (2011).

17. Chattopadhyay, M. K., Keembiyehetty, C. N., Chen, W. & Tabor, H. Polyamines stimulate the level of the σ^{38} subunit (RpoS) of *Escherichia coli* RNA polymerase, resulting in the induction of the glutamate decarboxylase-dependent acid response system via the *gadE* regulon. *J. Biol. Chem.* **290,** 17809–17821 (2015).
